# High Precision Dimensional Measurement with Convolutional Neural Network and Bi-Directional Long Short-Term Memory (LSTM)

**DOI:** 10.3390/s19235302

**Published:** 2019-12-02

**Authors:** Yuhao Wang, Qibai Chen, Meng Ding, Jiangyun Li

**Affiliations:** 1 School of Automation and Electrical Engineering, University of Science and Technology Beijing, Beijing 100083, China; b20140353@xs.ustb.edu.cn (Y.W.); 18811326398@163.com (Q.C.); 2 Key Laboratory of Knowledge Automation for Industrial Processes, Ministry of Education, Beijing 100083, China; 3 Scoop Medical, Houston, TX 77007, USA; meng.ding@okstate.edu

**Keywords:** dimensional measurement, sub-pixel edge detection, deep learning, convolutional neural network, bi-directional LSTM

## Abstract

In modern industries, high precision dimensional measurement plays a pivotal role in product inspection and sub-pixel edge detection is the core algorithm. Traditional interpolation and moment methods have achieved some success. However, those methods still have shortcomings. For example, the accuracy is still insufficient with the resolution limitation of the image sensor. Moreover, prediction results can be affected by image noise. With the recent success of deep learning technology, we propose a sub-pixel edge detection method based on convolution neural network (CNN) and bi-directional long short-term memory (LSTM). First, one-dimensional visual geometry group-16 (VGG-16) is employed to extract edge features. Then, a transformation operation is developed to generate sequence information. Lastly, bi-directional LSTM with fully-connected layers is introduced to output edge positions. Experimental results on our steel plate dataset demonstrate that our method achieves superior accuracy and anti-noise ability than traditional methods.

## 1. Introduction

**Figure 1:**
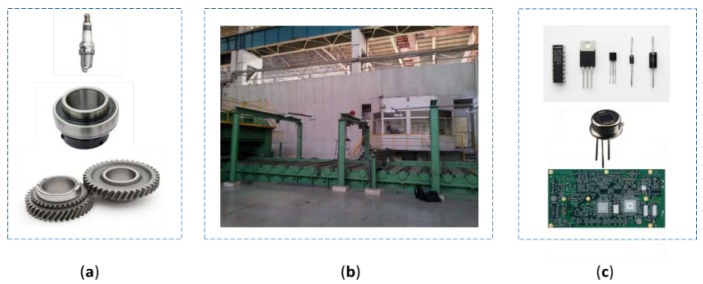
Applications of dimensional measurement with machine vision. (**a**) The parts of the automobile manufacturing industry. (**b**) The steel plate production line of iron and steel industry. (**c**) The electronic components and printed circuit board (PCB) of the electronic manufacturing industry.

A charge coupled device (CCD) is an important piece of digital imaging equipment. With the rapid development of CCD sensors and computer hardware, high precision dimensional measurement systems based on machine vision have been gradually adopted by industries such as automobile manufacturing, iron and steel manufacturing, and electronic manufacturing [1]. As shown in Figure 1, there are examples of industries applying dimensional measurement for their products. In the dimensional measurement systems, edge detection is the core algorithm. Due to the high precision requirements and the cost of the high-resolution CCD sensor, the accuracy of pixel-level edge detection is insufficient, and sub-pixel edge detection becomes an effective way to further improve the performance.

**Figure 2:**
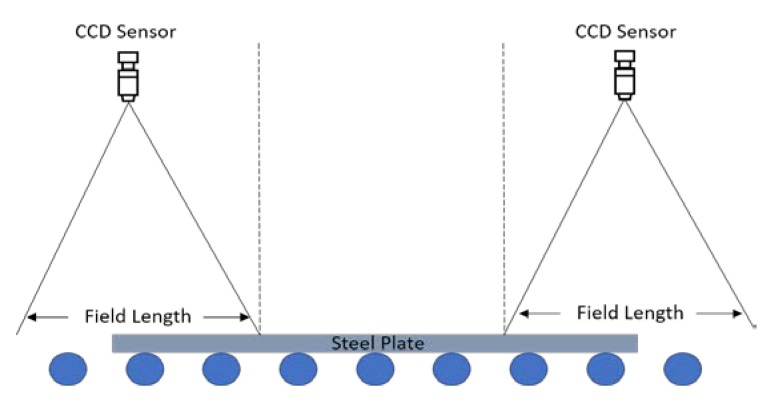
The principle diagram of the length measurement system of steel plate. CCD: charge coupled device.

In this work, we take the production line of steel plate in the Taiyuan iron and steel industry for research. Figure 1b shows the actual scene of the production line. Sheared steel plates are conveyed along with the roller; the aim of the task is to design a system for the measurement of length. Figure 2 shows the sketch map of the designed dimensional measurement system. Steel plate images are collected by two industrial-grade array CCD sensors and the length of the steel plate can be calculated by the sum of the field interval in the middle, and the steel plate’s length in the fields of two CCD sensors. Therefore, the core issue here is the high precision measurement of edge positions. Here, we take the images of one camera to test sub-pixel edge detection methods.

The concept of sub-pixel edge detection was first introduced by Hueckel [2], which includes three main methods: fitting method, interpolation method, and moment method. The fitting methods acquire edge location by fitting the grey value of the hypothetical edge model. The method proposed by Ye [3] adopts the Gaussian edge function obtained by convoluting the ideal edge model. The interpolation methods [4,5,6] interpolate the grey value of the pixel to increase information and locate the sub-pixel edge positions. Moreover, for the moment method, Ghosal first utilizes Zernike orthogonal moment on edge detection, which only needs three masks to be calculated [7,8,9,10]. The method proposed by Xie [11] improves the Zernike orthogonal moment with Roberts operator and Otsu’s method. However, in terms of the vast field-of-view of steel plates, those traditional methods cannot achieve a satisfactory accuracy with the resolution limitation of image. Furthermore, some images captured by the CCD sensor have noise such as scars and retro-reflective targets, which may affect the prediction results.

In recent years, the convolution neural network (CNN) has achieved great success in the computer vision field [12,13,14,15,16,17,18,19], including feature extraction, classification, and regression. CNN-based models learn network parameters directly from data using backpropagation and have more hidden layers which means a more powerful nonlinear fitting ability. In the early stage, CNN mainly focuses on classification tasks. LeNet was introduced by Yann in 1998 [20], which was designed to deal with the recognition of handwritten characters. After that, AlexNet [21], ZF-Net [22] and GoogleNet [23] were proposed at the ImageNet large scale visual recognition competition (ILSVRC), all of which achieved good grades. VGG was proposed by the visual geometry group of Oxford University at 2015 [24,25], which has more than 10 hidden layers, a smaller filter size, and a more robust feature extraction ability. However, image noise may affect the prediction results, while long short-term memory (LSTM) [26] has been proposed to handle the problem due to its excellent performance in analyzing sequence information.

LSTM is a widely used deep learning algorithm that aims to process and analyze sequence data. LSTM has been used in applications like natural language processing and the prediction of the stock market [27,28,29,30,31,32,33,34]. LSTM is developed based on the recurrent neural network (RNN) [35,36,37]. The traditional RNN has the problem of long-term dependency, which cannot connect the information when the gap between relevant information grows. LSTM avoids the long-term dependency problem by different gate structures that keep or drop out information. Moreover, the bi-directional LSTM model fuses forward propagation LSTM and backpropagation LSTM to connect both past and future information [38,39,40]. In our case, the positions of edge points in one image has relationships with both forward and backward ones. Therefore, bi-directional LSTM is a more appropriate option to optimize the edge positions further. For image noise, which may affect the extraction of edge points, bi-directional LSTM can learn edge information from adjacent unaffected edge points to rectify incorrect prediction results. Recent research has been proposed that combines the advantages of CNN and bi-directional LSTM for practical applications [41,42].

To further improve the accuracy of dimensional measurements with the limitation of image resolution, inspired by the analysis above, we propose a novel sub-pixel edge detection model based on CNN and bi-directional LSTM, which simultaneously has high precision and anti-noise ability. Our model adopts a one-dimensional visual geometry group-16 (VGG-16) to extract edge point features from the images. Then, a transformation module is developed to generate sequence information and bi-directional LSTM is followed to equip the model with anti-noise ability. In the end, a fully connected layer is employed to output the final prediction results. Experiments on our steel plate dataset demonstrate that the proposed model outperforms traditional methods and achieves only 0.112 of the overall mean absolute error (MAE) with the low image resolution of 512 pixels × 612 pixels.

The main contributions of this work are listed as follows:

1. We propose a sub-pixel edge detection method based on deep learning for high precision dimensional measurements.
2. We adopt CNN to extract features from images and introduce the anti-noise ability by adding bi-directional LSTM.
3. We offer a sub-pixel edge detection dataset of steel plate used in training and testing sub-pixel edge detection methods.

The remainder of the paper is organized as follows: Section 2 describes different components of the proposed sub-pixel edge detection system. Section 3 introduces the dataset, preprocessing methods, training protocol, and results. Section 4 is the discussion. Section 5 is the conclusion.

## 2. Methods

In this paper, we propose a sub-pixel edge detection method based on deep learning for high precision dimensional measurements. Our work aims to predict accurate edge positions of steel plates with the resolution limitation of image. In order to obtain low-resolution input data and the corresponding sub-pixel ground truth, two steps of preprocessing should be noticed. First, we downsample each image of steel plate by four times and collect 90 horizontal lines’ pixel value in the region of steel plate at the equal interval as the input data. Then, we measure the edge position of each line manually at the original resolution and divide the position by four to obtain the sub-pixel ground truth. Therefore, our method and comparison methods are all tested on the low-resolution images.

**Figure 3:**
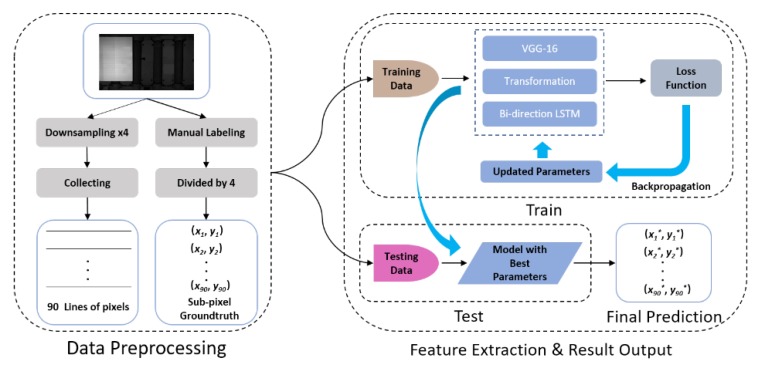
The pipeline of the proposed sub-pixel edge detection system, including data preprocessing, model training, and testing.

After the preprocessing steps, our proposed sub-pixel edge detection network is trained using the training data and the training procedure is based on the gradient descent algorithm which uses the updated parameters calculated by the loss function to improve the performance. Then, the trained model with the best parameters will be chosen to generate predictions on the test data. The pipeline of our sub-pixel edge detection system is illustrated in Figure 3.

### 2.1. Building Blocks

**Figure 4:**
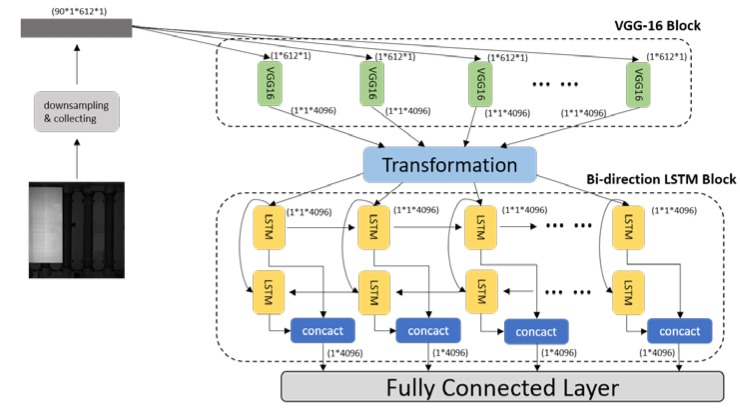
The architecture of the proposed model, consisting of preprocessing operation, visual geometry group-16 (VGG-16) block, transformation module, bi-directional long short-term memory (LSTM) block, and output fully connected layer.

In this section, we introduce the three building blocks in the proposed model. We first apply a one-dimensional VGG-16 to parallel extract edge features from the 90 collected lines of pixel value. Then, a transformation module is developed to generate sequence information. Lastly, bi-directional LSTM is employed to introduce the anti-noise ability and make the prediction results more accurate. The architecture of the proposed network is shown in Figure 4.

#### 2.1.1. VGG-16 as Feature Extractor

**Figure 5:**
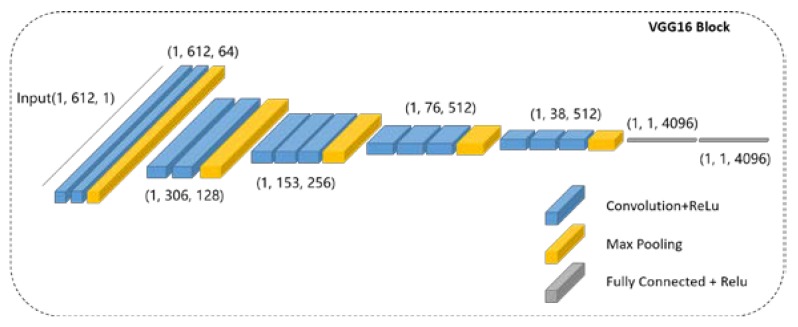
The structure of VGG-16 model used in this work, which consists of one-dimensional convolution layers, one-dimensional max-pooling layers, and fully connected layers.

The purpose of applying VGG-16 to our model is to extract edge features from the input lines of pixel values. VGG nets proved to have excellent feature extraction performances at the Imagenet large scale visual recognition competition in 2014. The increase in the depth of the convolution layers has a significant improvement on feature extraction and the application of small convolution kernels reduces the number of parameters. In this work, the inputs are 90 one-dimensional vectors. To extract edge features from them, we adopt a VGG-16 with the one-dimensional kernel in each convolution and max-pooling layer. The 90 input vectors are considered as a batch, and the one-dimensional VGG-16 can extract edge features parallel with the same weights. Figure 5 shows the details of the VGG-16 model used in this work.

#### 2.1.2. Transformation Module

A transformation module is developed to connect VGG-16 and bi-directional LSTM. In this work, 90 sets of data are calculated by VGG-16 and the output features are 90 one-dimensional vectors, while the input of bi-directional LSTM is the timing sequence with 90 moments. The transformation module converts the extracted features to sequence data. The process of the transformation operation is shown in Algorithm 1:


**Algorithm 1. The Process of the Transformation Operation**
1. All the outputs from VGG-16 are denoted as $V_{i}$, the form is (w,h,c), where w, h is the width and height of the feature map, and c is the number of channel. Expand the dimension of $V_{i}$ at first axis, the new form of $V_{i}$ is (1,w,h,c).2. Concat all the $V_{i}$ at first axis, the output is denoted as V, and the form is (N,w,h,c).3. Expand the dimension of V, the new form is (1,N,w,h,c).4. Compress the width, height, and channel to one dimension, the form of the final output is (1,N,w*h*c).

#### 2.1.3. Bi-Directional LSTM

**Figure 6:**
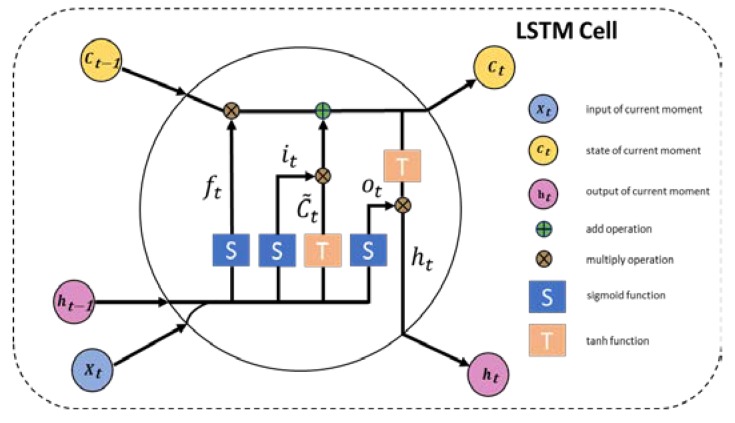
The structure of the LSTM cell. Different gates in the LSTM cell will decide which information to keep and drop out, and the cell state is updated.

The bi-directional LSTM network is introduced to improve accuracy and add anti-noise ability. The LSTM network has excellent performance in handling context information, while bi-directional LSTM not only accesses past context information but can also obtain future context information. In this work, the 90 edge points in one image have logical connections and their values vary little from both forward and backward ones. Therefore, bi-directional LSTM is more appropriate for our task compared to the vanilla LSTM. Figure 6 shows the details of the LSTM cell. The input of LSTM is the timing sequence, and for the input at any moment, there are three steps in a LSTM cell.

The first step is to decide which context information to throw away from the cell state of last moment by a sigmoid layer called forget gate layer. $f_{t}$ decides which information to throw off and $f_{t}$ is formulated as follow:

(1) $$f_{t}=\sigma (W_{f}\cdot [h_{t-1},x_{t}]+b_{f})$$

where $W_{f}$ is the weight of forget gate layer, $b_{f}$ is the bias, and σ is sigmoid operation. Then, another sigmoid layer called input gate layer is applied to decide which information should be reserved. $i_{t}\cdot {\tilde{C}}_{t}$ decides which information to keep, and the expression of $i_{t}$ and ${\tilde{C}}_{t}$ are as follows:

(2) $$i_{t}=\sigma (W_{i}\cdot [h_{t-1},x_{t}]+b_{i})$$

(3) $${\tilde{C}}_{t}=\tanh(W_{C}\cdot [h_{t-1},x_{t}]+b_{C})$$

where $W_{i}$ and $W_{C}$ is the weight of the input gate layer, $b_{i}$ and $b_{C}$ is the bias. Lastly, the third step is to decide which information deserved to be output, and this layer is called output gate layer. $h_{t}$ decides which information to output and the expressions of $h_{t}$ are as follows:

(4) $$o_{t}=\sigma (W_{o}\cdot [h_{t-1},x_{t}]+b_{o})$$

(5) $$h_{t}=o_{t}*\tanh(C_{t})$$

With these three steps, the proposed model will obtain anti-noise ability and have more accurate predictions. The detailed configurations of the network are shown in Table 1.

### 2.2. MSE as Loss Function

The loss function is a necessary component in the deep learning network to calculate the deviation value between the prediction and ground truth and optimize the network parameters through backpropagation. In this work, we adopt the mean square error (MSE) loss function at the end of the proposed network. The expression of MSE is:

(6) $$MSE=\frac{1}{m}\sum_{i=1}^{m}{\left({\hat{X}}_{i}-X_{i}\right)}^{2}$$

where ${\hat{X}}_{i}$ is the predicted value, $X_{i}$ is the ground truth, and m is the batch size. During the training phase, the proposed network is trained by the stochastic gradient descent (SGD) algorithm to minimize the MSE loss.

## 3. Results

### 3.1. Dataset

**Figure 7:**
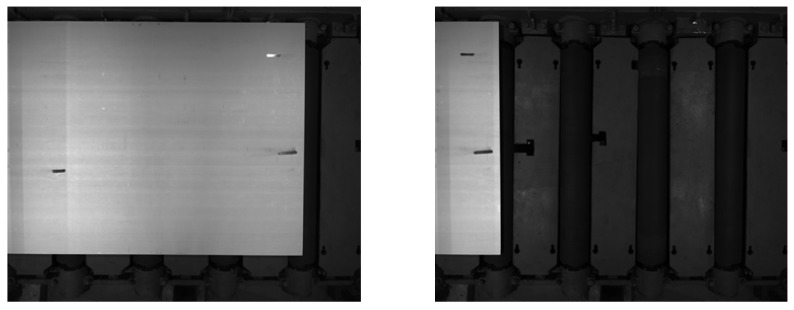
Samples of steel plate images captured by the industrial-grade array CCD sensor. The steel plate is moving through the roller.

We evaluate our sub-pixel edge-detection system on the steel plate images collected at the Taiyuan Iron and Steel industry. At the end of the production line, all the finished steel plates need to be measured by the system. The dataset is captured by the industrial-grade array CCD sensor in the stainless-steel cold rolling production line with roller and steel plate in the image. The type of CCD sensor is Point Grey FL3-U3-120S3C-C and the resolution is 2048×2448. All the images have corresponding ground truth, 241 images for training, and 62 images for testing. Figure 7 shows the samples of the dataset.

### 3.2. Preprocessing the Dataset

To verify the superiority of the proposed method with the limitation of image resolution, all the images in the dataset need to be preprocessed. It is unnecessary to calculate all edge positions for length measurement and getting a fixed number of edge positions at equal interval is sufficient. Therefore, the preprocessing includes two steps: downsampling and collecting one-dimensional horizontal vectors from each image.

First, we downsample the original images by four times to obtain low-resolution images with the size of 512×612. Then, we select the region of interest (ROI) that covers the steel plate and pick 90 one-dimensional horizontal vectors at equal interval as the input data. The resolution of each vector is 1×612.

Moreover, different from pixel-level edge detection, it is impossible to obtain absolute ground truth of sub-pixel edge position. Thus, the edge position of each selected vector is calculated manually on the original images and divided by four. In this way, the error of ground truth is within one-fourth of a pixel, and the accuracy can be guaranteed to the greatest extent. Finally, every 90 selected vectors and the corresponding ground truth from one image are considered as one set of input data to the proposed model.

### 3.3. Training Protocol and Metrics

The proposed sub-pixel edge detection model is deployed on Google Tensorflow deep learning platform with one NVIDIA GTX1080Ti GPU (11GB RAM). During the training procedure, the learning rate starts with 0.0001 and decays 5% every 241 iterations. The total number of iterations is 80,000.

The metrics to evaluate the proposed sub-pixel edge detection model involve three different criteria: mean-absolute error (MAE), MSE, and root mean square error (RMSE). The formulas are as follows:

(7) $$MAE=\frac{1}{n}\sum_{i=1}^{n}\left|x_{i}-m(x)\right|$$

(8) $$MSE=\frac{1}{n}{\sum_{i=1}^{n}\left(x_{i}-m(x_{i})\right)}^{2}$$

(9) $$RMSE=\sqrt{\frac{1}{n}{\sum_{i=1}^{n}\left(x_{i}-m(x_{i})\right)}^{2}}$$

where n is the total number of edge points, $m(x_{i})$ is the prediction, and $x_{i}$ is the ground truth.

### 3.4. Experimental Results

To better evaluate the proposed sub-pixel edge detection model, traditional methods including interpolation method and moment method are adopted as the baseline for comparison. For the interpolation method, we introduce the quadratic interpolation algorithm, while for the moment method, the Sobel-Zernike operator is employed.

**Figure 8:**
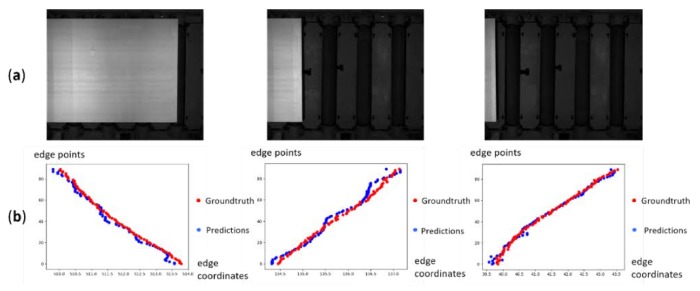
Samples of images and the prediction results. (**a**) The images captured by the CCD sensor. (**b**) The plots of the predictions: blue dots represent the predictions from our model and red dots represent the corresponding ground truth.

Table 2 shows the detail results of the proposed model on our steel plate dataset. We evaluate the model based on the four times downsampled images and Figure 8 shows examples of the prediction results. The first row is the steel plate images; the second row is the predictions and the corresponding ground truth, where the vertical coordinate represents the serial number of the edge points, and the horizontal ordinate represents the coordinate of the edge point.

## 4. Evaluation and Discussion

### 4.1. The Selection of Feature Extraction Block

CNN based models are popular in extracting image features, and numerous CNN-based models such as AlexNet, VGG, ResNet, and DenseNet have been proposed in the competition of ImageNet since 2012 with excellent results.

In the proposed model, it is appropriate to apply VGG-16 as the feature extraction block and in order to prove the superiority of VGG-16 in extracting edge point features, we also explore VGG-19, ResNet, and DenseNet as comparison. Table 3 shows the details of the comparison. As can be seen, equipping feature extraction block with VGG-16 has less error in prediction than other CNN-based models.

### 4.2. The Effect of the Fully Connected Layers in VGG-16

In this work, we adopt a one-dimensional VGG-16 with the standard structure as the feature extractor and there are two fully connected layers with 4096 output channels in the end. Different from the convolution layer, the fully connected layer does not share weights. To evaluate the effect of the fully connected layers, we did comparative experiments with two additional models. In the first model, the two fully connected layers are replaced by one convolution layer with the kernel size of 1×38 and the output channels of 4096, while the second model changed the output channels of the two fully connected layers to 2048. As shown in Table 4, our model has the best accuracy. The reason may come from two parts. The characteristics of shared weights reduce the nonlinear fitting ability of convolution layer and fewer channels hinder the feature extraction.

### 4.3. The Importance of Bi-Directional LSTM

LSTM is an effective model to deal with sequence information, while bi-directional LSTM has the ability to process information at the present moment according to both the past and future information. This characteristic makes bi-directional LSTM can optimize the edge positions further and has the anti-noise ability. To better evaluate the effects of adding bi-directional LSTM into our model, we did additional experiments compared to the model with vanilla LSTM and the model without any types of LSTM. As shown in Table 5, the proposed model with bi-directional LSTM achieves the best accuracy and the relative error of the predictions from the proposed model is 40% lower than the model without any types of LSTM. The results demonstrate that bi-directional LSTM is effective and performs better than vanilla LSTM.

**Figure 9:**
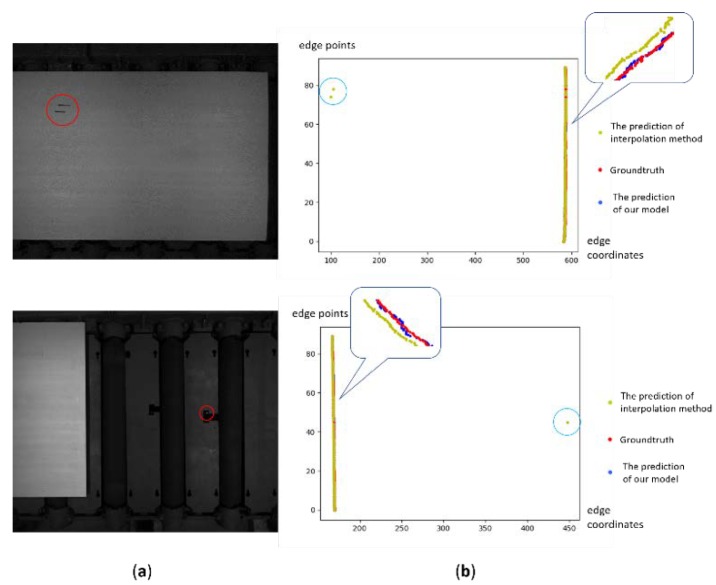
Samples of the prediction from our model on the images with image noise. (**a**) The images captured by the CCD sensor. (**b**) The plots of predictions. Our model predicts more accurate edge point positions without being affected by image noise.

For the edge points which may be influenced by image noise in this work, the proposed model can rectify the prediction results according to the context information. Figure 9 shows the examples of predictions results with image noise. The first column is the images with noise such as scars and retro-reflective targets, and the second column represents the predictions from the proposed model and interpolation method. For the areas with noise, the interpolation method incorrectly considers the noise point as the edge point, while the proposed model is not affected by the noise.

The comparison above indicates that the bi-directional LSTM can reduce the effect of image noise and is also helpful to improve the accuracy of predictions.

### 4.4. The Comparison to Other Methods

There are three main sub-pixel edge detection methods: the fitting method, interpolation method, and moment method. There is also the interpolation method with quadratic interpolation algorithm and the moment method with Sobel-Zernike operator, which are widely used in the sub-pixel edge detection. In order to evaluate the proposed dimensional measurement model, we apply those two methods as comparison.

**Figure 10:**
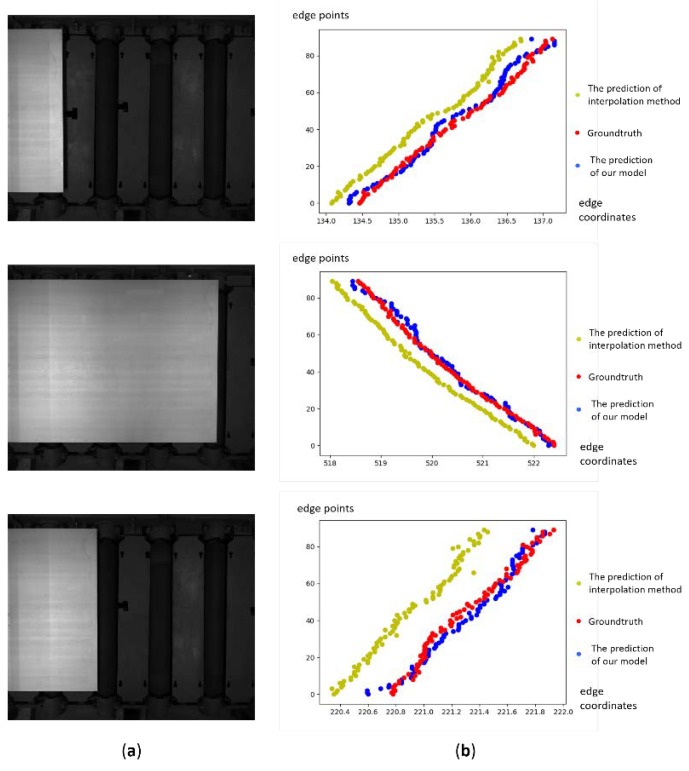
Samples of comparison result from our model and the interpolation method. (**a**) The images captured by the CCD sensor. (**b**) The plots of the comparison results.

Figure 10 shows the samples of the comparison between quadratic interpolation method and our model, where the first column is the steel plates images captured by the CCD sensor; the second column is the ground truth and the predictions from different methods.

Results on our steel plates dataset (Table 6) demonstrate that the proposed model has superior improvement on the accuracy of prediction than other methods.

## 5. Conclusions

In this paper, we propose a novel network based on CNN and bi-directional LSTM to perform sub-pixel edge detection on steel plate images. The main contribution of this work includes introducing one-dimensional VGG-16 to extract edge features and applying bi-directional LSTM to handle sequence information. The purpose of our work is to find a way to locate edge positions on steel plate images more accurately with the limitation of resolution and has anti-noise ability. The comparison between traditional sub-pixel methods indicates that our method achieves significant improvement than other existed methods.

## Figures and Tables

**Table 1:** Configurations of the proposed network.

	Layer	Type	Kernel Size	Resolution
VGG-16	conv1_1	convolution	1 × 3, 64	1 × 612
	conv1_2	convolution	1 × 3, 64	1 × 612
	pool_1	max_pooling	1 × 2, 64, stride 2	1 × 306
	conv2_1	convolution	1 × 3, 128	1 × 306
	conv2_2	convolution	1 × 3, 128	1 × 306
	pool_2	max_pooling	1 × 2, 128, stride 2	1 × 153
	conv3_1	convolution	1 × 3, 256	1 × 153
	conv3_2	convolution	1 × 3, 256	1 × 153
	conv3_3	convolution	1 × 3, 256	1 × 153
	pool_3	max_pooling	1 × 2, 256, stride 2	1 × 76
	conv4_1	convolution	1 × 3, 512	1 × 76
	conv4_2	convolution	1 × 3, 512	1 × 76
	conv4_3	convolution	1 × 3, 512	1 × 76
	pool_4	max_pooling	1 × 2, 512, stride 2	1 × 38
	conv5_1	convolution	1 × 3, 512	1 × 38
	conv5_2	convolution	1 × 3, 512	1 × 38
	conv5_3	convolution	1 × 3, 512	1 × 38
	pool_5	max_pooling	1 × 2, 512, stride 2	1 × 19
	fc_1	fully_connected	-, 4096	-
	fc_2	fully_connected	-, 4096	-
Bi-directional LSTM	block1	-	-, 4096	-
Output	fc_3	fully_connected	-, 90	-

**Table 2:** The metric scores of mean-absolute error (MAE), mean square error (MSE), and root mean square error (RMSE) from the proposed model.

Method	MAE	MSE	RMSE
Proposed model (VGG-16 + Bi-LSTM)	0.112	0.0406	0.202

**Table 3:** The comparison of VGG-16 with other feature extraction blocks.

Method	MAE	MSE	RMSE
VGG-19 + Bi-LSTM	0.121	0.0436	0.209
ResNet + Bi-LSTM	0.124	0.0438	0.209
DenseNet + Bi-LSTM	0.117	0.0450	0.212
Proposed Model (VGG-16 + Bi-LSTM)	0.112	0.0406	0.202

**Table 4:** The comparison results of fully connected layer with convolution layer. FC: fully connected layer; Conv: convolution layer.

Method	MAE	MSE	RMSE
Conv with 4096	0.118	0.0423	0.206
FC with 2048	0.116	0.0418	0.204
Ours (FC with 4096)	0.112	0.0406	0.202

**Table 5:** The comparison results of our model with vanilla LSTM, bi-directional LSTM, or without any of them.

**Model**	**VGG-16**	**Vanilla LSTM**	**Bi-directional LSTM**	**MAE**	**MSE**	**RMSE**
	√	×	×	0.183	0.0610	0.247
	√	√	×	0.134	0.0482	0.220
	√	×	√	0.112	0.0406	0.202

**Table 6:** The quantitative results of different methods.

Method	MAE	MSE	RMSE
Quadratic Interpolation Method [5]	0.378	0.151	0.389
Moment Method with Sobel-Zernike operator [10]	0.355	0.149	0.386
Proposed model (VGG-16 + LSTM)	0.112	0.0406	0.202

## References

[1] Pangrazio J.G., Pangrazio J.A., Pangrazio R.T., Brey K.L., Pena-Gutierrez C. (2012). Dimensional Detection System and Associated Method. U.S. Patent.

[2] Lyvers E., Mitchell O., Akey M., Reeves A. (1989). Subpixel measurements using a moment-based edge operator. IEEE Trans. Pattern Anal. Mach. Intell..

[3] Wang Y.P., Ye A. (1999). Sub-Pixel Dataform Reader with Dynamic Noise Margins. U.S. Patent.

[4] Rösgen T. (2003). Optimal subpixel interpolation in particle image velocimetry. Exp. Fluid..

[5] Nalwa V.S. (1987). Edge-Detector Resolution Improvement by Image Interpolation. IEEE Trans. Pattern Anal. Mach. Intell..

[6] Pap L., Zou J.J. Sub-pixel edge detection for photogrammetry using laplace difference of Gaussian and 4th order ENO interpolation. Proceedings of the 2010 IEEE International Conference on Image Processing.

[7] Ghosal S., Mehrotra R. (1994). Detection of composite edges. IEEE Trans. Image Process..

[8] Ghosal S., Mehrotra R. (1993). Orthogonal moment operators for subpixel edge detection. Pattern Recognit..

[9] Da F., Zhang H. (2010). Sub-pixel edge detection based on an improved moment. Image Vis. Comput..

[10] Yang H., Pei L. Fast algorithm of subpixel edge detection based on Zernike moments. Proceedings of the 2011 4th International Congress on Image and Signal Processing.

[11] Xie X., Ge S., Xie M., Hu F., Jiang N. (2019). An improved industrial sub-pixel edge detection algorithm based on coarse and precise location. J. Ambient. Intell. Humaniz. Comput..

[12] Krizhevsky A., Sutskever I., Hinton G.E. (2017). Pdf ImageNet classification with deep convolutional neural networks. Commun. ACM.

[13] Karpathy A., Toderici G., Shetty S., Leung T., Sukthankar R., Fei-Fei L., Shetty S., Leung T. Large-Scale Video Classification with Convolutional Neural Networks. Proceedings of the 2014 IEEE Conference on Computer Vision and Pattern Recognition.

[14] Hu B., Lu Z., Li H., Chen Q. Convolutional Neural Network Architectures for Matching Natural Language Sentences. Proceedings of the International Conference on Neural Information Processing Systems.

[15] Rastegari M., Ordonez V., Redmon J., Farhadi A. (2016). XNOR-Net: ImageNet Classification Using Binary Convolutional Neural Networks. European Conference on Computer Vision.

[16] Zhang X., Zhou X., Lin M., Sun J. ShuffleNet: An Extremely Efficient Convolutional Neural Network for Mobile Devices. Proceedings of the 2018 IEEE/CVF Conference on Computer Vision and Pattern Recognition.

[17] Lawhern V.J., Solon A.J., Waytowich N.R., Gordon S.M., Hung C.P., Lance B.J. (2018). EEGNet: A compact convolutional neural network for EEG-based brain–computer interfaces. J. Neural Eng..

[18] Jin K.H., McCann M.T., Froustey E., Unser M. (2017). Deep Convolutional Neural Network for Inverse Problems in Imaging. IEEE Trans. Image Process..

[19] Chen H., Zhang Y., Zhang W., Liao P., Li K., Zhou J., Wang G. (2017). Low-dose CT via convolutional neural network. Biomed. Opt. Express.

[20] LeCun Y., Bottou L., Bengio Y., Haffner P. (1998). Gradient-based learning applied to document recognition. Proc. IEEE.

[21] Xiao L., Yan Q., Deng S. Scene classification with improved AlexNet model. Proceedings of the 2017 12th International Conference on Intelligent Systems and Knowledge Engineering (ISKE).

[22] Zeiler M.D., Fergus R. (2014). Visualizing and Understanding Convolutional Networks. European Conference on Computer Vision.

[23] Szegedy C., Liu W., Jia Y., Sermanet P., Reed S., Anguelov D., Erhan D., Vanhoucke V., Rabinovich A. Going deeper with convolutions. Proceedings of the IEEE Conference on Computer Vision and Pattern Recognition.

[24] Simonyan K., Zisserman A. (2014). Very Deep Convolutional Networks for Large-Scale Image Recognition. arXiv.

[25] Sun Y., Liang D., Wang X., Tang X. (2015). DeepID3: Face Recognition with Very Deep Neural Networks. arXiv.

[26] Hochreiter S., Schmidhuber J. (1997). Long short-term memory. Neural Comput..

[27] Greff K., Srivastava R.K., Koutník L., Steunebrink B.R., Schmidhuber J. (2017). LSTM: A Search Space Odyssey. IEEE Trans. Neural Netw. Learn. Syst..

[28] Shi X., Chen Z., Wang H., Yeung D.-Y., Wong W.-K., Woo W.-C. Convolutional LSTM Network: A Machine Learning Approach for Precipitation Nowcasting. Proceedings of the International Conference on Neural Information Processing Systems.

[29] Huang Z., Xu W., Yu K. (2015). Bidirectional LSTM-CRF Models for Sequence Tagging. arXiv.

[30] Ordóñez F.J., Roggen D. (2016). Deep Convolutional and LSTM Recurrent Neural Networks for Multimodal Wearable Activity Recognition. Sensors.

[31] Chiu J.P., Nichols E. (2016). Named Entity Recognition with Bidirectional LSTM-CNNs. Trans. Assoc. Comput. Linguist..

[32] Wu Y., Yuan M., Dong S., Lin L., Liu Y. (2018). Remaining useful life estimation of engineered systems using vanilla LSTM neural networks. Neurocomputing.

[33] Ma C.-Y., Chen M.-H., Kira Z., AlRegib G. (2019). TS-LSTM and temporal-inception: Exploiting spatiotemporal dynamics for activity recognition. Signal Process. Image Commun..

[34] Zhang P., Ouyang W., Zhang P., Xue J., Zheng N. (2019). SR-LSTM: State Refinement for LSTM towards Pedestrian Trajectory Prediction. arXiv.

[35] Paliwal K., Schuster M. (1997). Bidirectional recurrent neural networks. IEEE Trans. Signal Process..

[36] Graves A., Mohamed A.-R., Hinton G., Graves A. Speech recognition with deep recurrent neural networks. Proceedings of the 2013 IEEE International Conference on Acoustics, Speech and Signal Processing.

[37] Zaremba W., Sutskever I., Vinyals O. (2014). Recurrent Neural Network Regularization. arXiv.

[38] Chen T., Xu R., He Y., Wang X. (2017). Improving sentiment analysis via sentence type classification using BiLSTM-CRF and CNN. Expert Syst. Appl..

[39] Luo L., Yang Z., Yang P., Zhang Y., Wang L., Lin H., Wang J. (2017). An attention-based BiLSTM-CRF approach to document-level chemical named entity recognition. Bioinformatics.

[40] Talman A., Yli-Jyrä A., Tiedemann J. (2018). Natural Language Inference with Hierarchical BiLSTM Max Pooling Architecture. arXiv.

[41] Chen Z., Zhao R., Zhu Q., Masood M.K., Soh Y.C., Mao K. (2017). Building occupancy estimation with environmental snesors via CDBLSTM. IEEE Trans. Ind. Electron..

[42] Liu X., Liu Y., Zhang M., Chen X., Li J. (2019). Improving Stockline Detection of Radar Sensor Array Systems in Blast Furnaces Using a Novel Encoder–Decoder Architecture. Sensors.

